# Pancreatic follicular dendritic cell sarcoma: a rare case report and systematic literature review of 7 cases

**DOI:** 10.1186/s12957-023-03115-5

**Published:** 2023-07-21

**Authors:** Xu Li, Jin Gu, Qingyun He, Shuwen Han, Huichao Wu

**Affiliations:** 1 Department of Gastroenterology, Digestive Disease Hospital, The Affiliated Hospital of Zunyi Medical University, Zunyi City, Guizhou Province People’s Republic of China; 2 Department of General Surgery, The Affiliated Hospital of Zunyi Medical University, Zunyi City, Guizhou Province People’s Republic of China

**Keywords:** Follicular dendritic cell sarcoma, Pancreas, CT, MRI, Diagnosis, Treatment

## Abstract

**Introduction:**

Pancreatic follicular dendritic cell sarcoma (FDCS) is an exceptionally rare and low-to-moderate malignancy, with only seven reported cases to date. Clinical diagnosis of FDCS is challenging due to the lack of distinct biological and radiographic features.

**Case presentation:**

A 67-year-old woman presented to the hospital with a 4-day history of severe abdominal pain. Imaging studies (CT and MRI) revealed a large cystic mass located at the tail of the pancreas, which was suspected to be myeloid sarcoma (MS) based on EUS and CT-guided pancreatic puncture. Postoperative pathology and immunohistochemistry confirmed the diagnosis of pancreatic FDCS. After the diagnosis was confirmed, the patient received postoperative chemotherapy with the CHOP regimen. At 11 months of follow-up, there was no evidence of recurrence. Seven published cases have been reviewed to comprehensively summarize the clinical characteristics, diagnosis, and treatment options of FDCS.

**Conclusion:**

While imaging can be useful in detecting pancreatic FDCS, it should be interpreted with caution as it can be challenging to differentiate from other pancreatic tumors. Pathology and immunohistochemistry are considered the gold standard for diagnosis, with CD21, CD23, and CD35 being specific tumor cell markers. However, preoperative diagnosis of pancreatic FDCS remains difficult, and the pancreatic puncture may further increase the risk of misdiagnosis. The disease is highly prone to recurrence and metastasis, and surgery is the preferred method for both diagnosis and treatment of localized disease.

## Introduction

Follicular dendritic cell sarcoma (FDCS) was first discovered and named by Monda et al. in 1986 [1]. FDCS is a malignant tumor that originates from follicular dendritic cells, mainly affecting the head and neck lymph nodes and manifesting as a slow-growing mass. Approximately, one-third of cases can extensively involve extranodal organs including bone, liver, spleen, skin, and soft tissue [2]. While the abdomen is the most common extranodal site [3], pancreatic FDCS is extremely rare, with only seven cases reported to date (Table 1). The male-to-female ratio is 4:3, and the age of onset varies from 30 to 70 years old. The clinical symptoms of the disease are atypical, and in previous reports, only one case was associated with rare myasthenia gravis and para tumor pemphigus [4], while the rest were not specific. This article presents a case of primary pancreatic FDCS and provides a comprehensive review of the literature to enhance clinicians’ understanding of the onset.

**Table 1:** FDCS in the pancreatic have been previously reported cases in addition to present case

Publication year	Age/sex	Country	Location	Size (cm)	Immunohistochemistry	Treatment	Follow-up/outcome	Metastasis
1995 [5]	63/M	UK	Head	15 × 11.5 × 9.5	CD21, CD35	Surgery	4 months/NR	None
2006 [6]	64/M	China	Head	10.5 × 9.0 × 6.5	CD21, CD23, CD35	Surgery	18 months/R	Liver
2007 [7]	56/M	USA	-	2	CD21, CD23	Surgery XRT + CHOP	7 months/R	LN
2016 [8]	67/F	China	Tail	4 × 4 × 4.5	CD21, CD23	Surgery	-	None
2019 [4]	49/F	China	Tail	4 × 5	CD21, CD23, CD35	Surgery	15 days/death	None
2019 [9]	70/F	USA	Tail	8.5 × 5.5 × 3.2	CD21, CD23, CD35	Surgery	3 months/R	Spleen
2022 [10]	30/M	China	Head	10 × 9 × 8	CD21, CD35	Surgery	3 months/R	Liver, LN
2023	67/M	China	Tail	6 × 5	CD21, CD35	Surgery	8 months/R	Liver, LN

*M* male, *F* female, *R* recurrence, *NR* no recurrence, *LN* lymph node, *RTX* radiation, *CHOP* cyclophosphamide doxorubicin vincristine dexamethasone. -This content was not described

## Case presentation

**Fig. 1:**
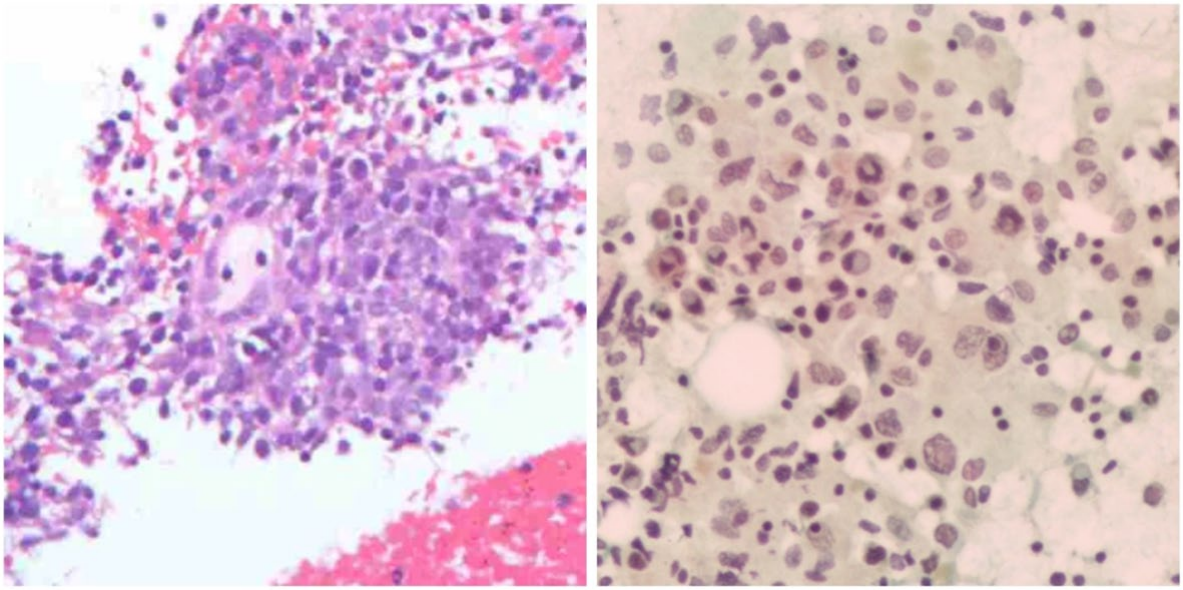
EUS-guided pancreatic puncture. **a** HE staining of tissue sample showing small round cells with focal distribution. **b** Tissue smear showing a small number of degenerated dystopic cells

**Fig. 2:**
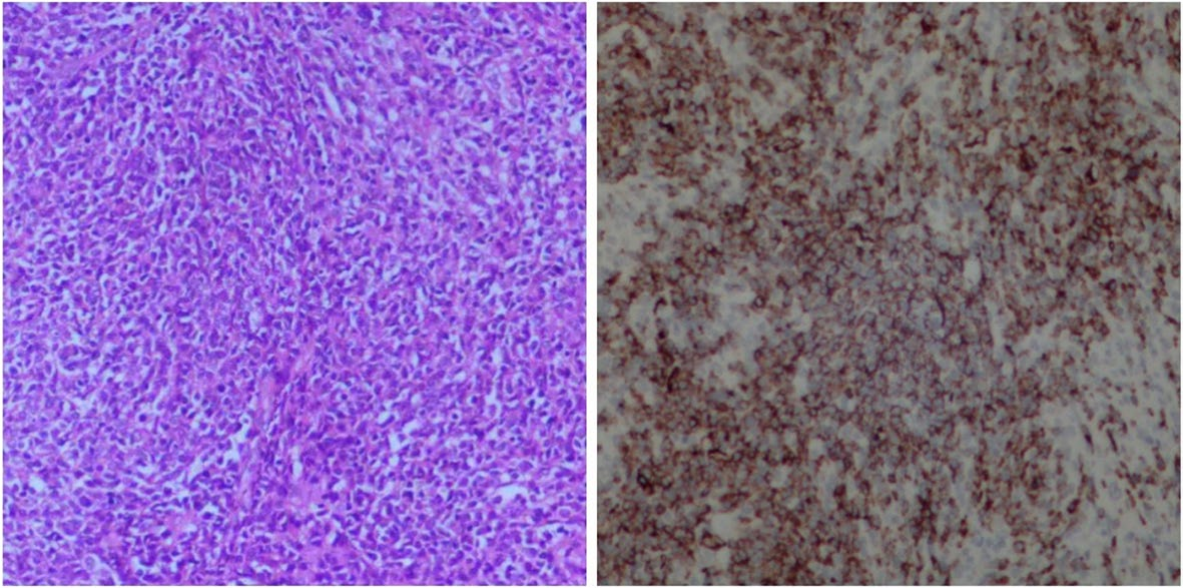
CT-guided pancreatic puncture suspected myeloid sarcoma

**Fig. 3:**
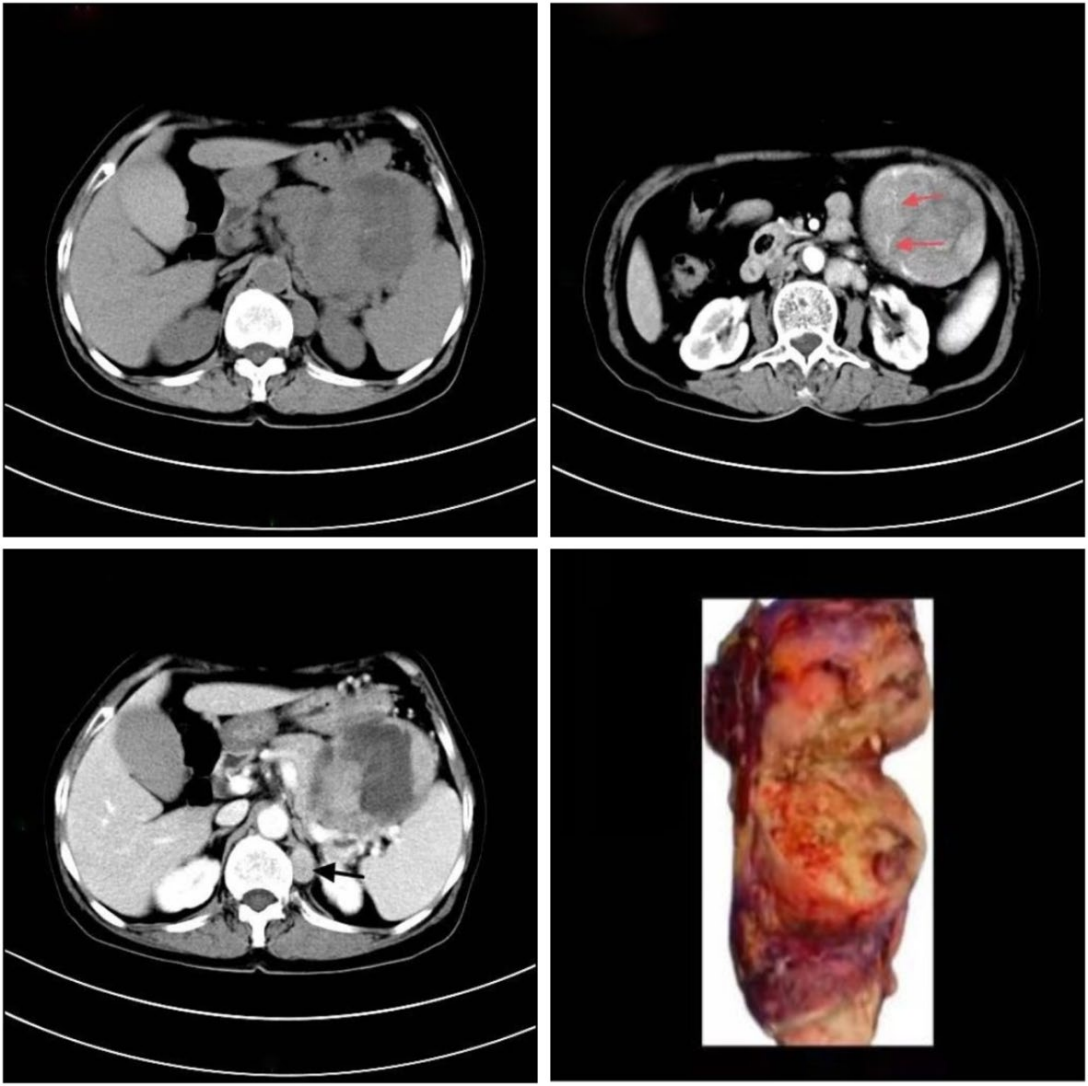
CT image of pancreatic FDCS. **a** Plain CT scan shows pancreatic caudal round cystic masses, about 8.8 cm × 8.4 cm × 9 cm, with uneven density, and patchy, slightly high-density shadows. **b**–**c** Enhanced scan showing the solid part of the tumor being strengthened, enhanced scan continues to be strengthened, with the blood supply artery visible around the tumor (red arrow); the cystic area is not strengthened, and the abdominal lymph nodes are enlarged (black arrow). **d** General specimen: tumor texture is medium, capsule is complete, dark red fish-like appearance

**Fig. 4:**
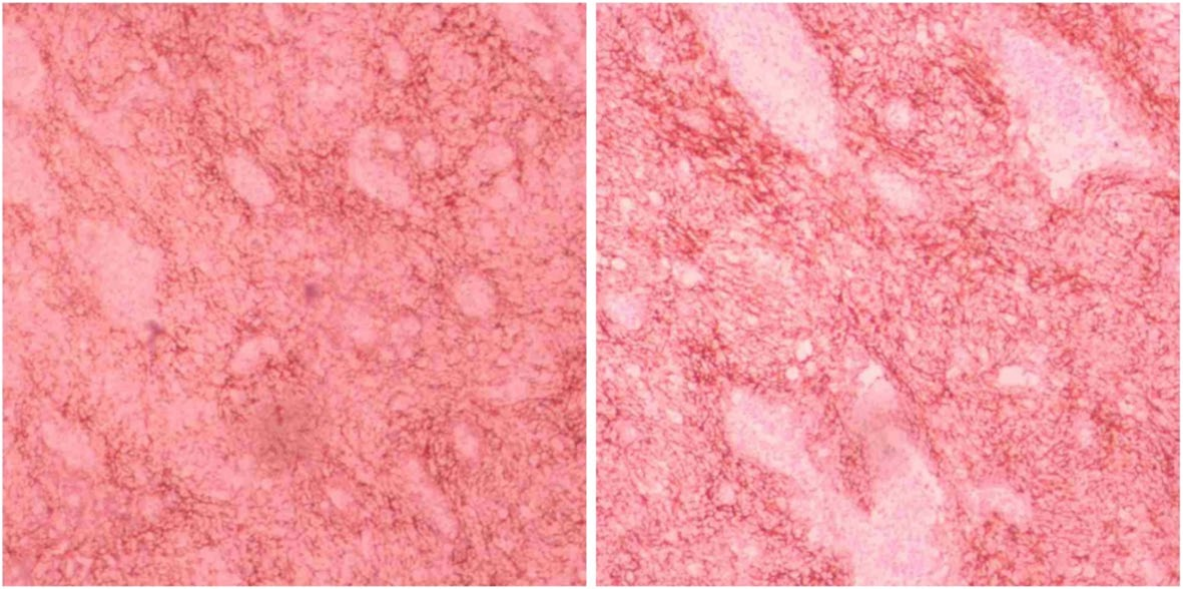
Pancreatic FDCS confirmed after surgery. **a** Immunohistochemical staining showing positive expression CD21. **b** Immunohistochemical staining showing positive expression CD35

A 67-year-old woman presented to our hospital with a 4-day history of abdominal pain. On physical examination, marked tenderness was noted in the left upper quadrant without rebound tenderness or muscle tension, and there was no palpable superficial lymphadenopathy. Laboratory tests revealed a hemoglobin level of 90 g/L, while the liver and kidney function, amylase, alpha-fetoprotein, CA199, CA125, and CEA were within normal ranges. Computed tomography (CT) and magnetic resonance imaging (MRI) showed solid mass lesions in the caudal pancreatic region and cystic on the large curvature of the stomach, with poorly demarcated margins from surrounding tissues and liquefied necrosis in the center of the lesion. Based on these findings, the preliminary diagnosis was gastrointestinal stromal tumors (GIST) or pancreatic neuroendocrine tumors (pNENs). To confirm the nature of the mass, an EUS-guided pancreatic puncture was performed, but the obtained material was insufficient for a definitive diagnosis (Fig. 1a–b). The patient underwent immunohistosis which suspected myeloid sarcoma (MS) with CT-guided pancreatic puncture (Fig. 2a–b), but no abnormalities were detected in bone marrow examination. Finally, the multidisciplinary team performed an “open tail pancreatic resection + total splenectomy.” During the operation, masses measuring 6 cm × 5 cm and 10 cm × 8 cm were found at the tail of the pancreas and near the spleen respectively. The capsule was complete, and the masses had a dark red fish-like appearance (Fig. 3d). Immunohistochemical analysis (Fig. 4a–b) revealed that tumor cells expressed CD21, CD35, and Ki67, while EBER, S-100, and CD117 were all negative. These findings confirmed that the patient had FDCS of the pancreas, which did not invade the spleen. Following the surgery, the patient received chemotherapy with the CHOP regimen (cyclophosphamide + doxorubicin + vincristine + dexamethasone). At an 11-month follow-up, no recurrence was observed.

## Discussion

**Fig. 5:**
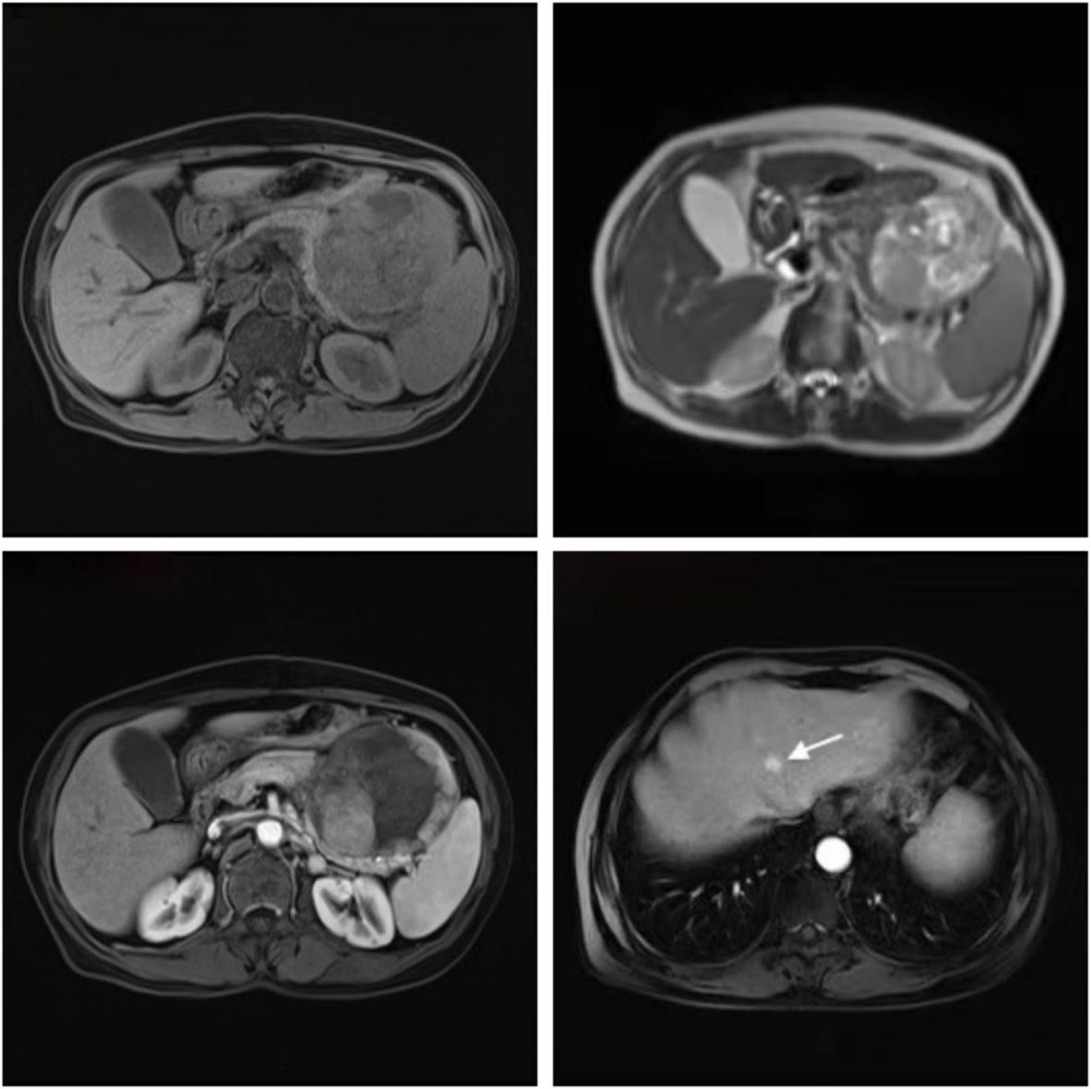
MRI image of pancreatic FDCS. **a** MRI scan showing a mass at the tail of the pancreas, with T1WI mixed signal mainly isosignal. **b** T2WI showing slightly higher signal. **c** Enhanced scan showing uneven and significant strengthening and the cystic region remaining unenhanced. **d** Multiple persistent strengthening nodules in the liver (white arrows)

FDCS in the abdomen is typically identified on imaging as a heterogeneous soft-tissue mass with complex internal structures, local necrosis, and lymphadenopathy. Tissue calcification may also be present, and contrast-enhanced scanning often reveals a “rapid wash-in and slow wash-out” or “progressive enhancement” [11–13]. However, due to the rarity of pancreatic FDCS, imaging features of this form of the disease have been reported only in a limited number of cases. Of the four cases currently evaluated, two occurred at the head of the pancreas, leading to significant bile duct dilation due to mass compression [6, 10]. The other two cases occurred at the tail of the pancreas and were characterized with a heterogeneous pattern of enhancement solid parts and enhanced vascular shadows within the mass, suggesting a relatively rich blood supply [4, 8]. The non-contrast CT scan of patient (Fig. 3a) revealed the presence of a cystic mass lesion measuring 8.8 cm × 8.4 cm × 9 cm in the pancreatic tail area, adjacent to the large curvature of the stomach and spleen. The tumor showed compression of surrounding tissues, with evidence of bleeding necrosis in the central pancreatic tail. The solid part of the enhanced scan showed continuous strengthening, while the cystic area showed no strengthening (Fig. 3b–c). Blood supply arteries around the tumor were also visible. MRI examination showed abnormal signals of the pancreatic tail (Fig. 5a–d), with the T1-weighted imaging (T1WI) scan dominated by isosignal and the T2-weighted imaging (T2WI) scan showing a slightly higher signal shadow. The solid part of the enhanced scan was unevenly and significantly strengthened, while the cystic area was not enhanced. Multiple progressive strengthening nodules and enlarged lymph nodes were observed in the liver and were finally confirmed as metastatic lesions by tomography (PET-CT). Based on the imaging features mentioned above, pNENs and GITS were initially considered in our patient. The pancreas, being a rare site for FDCS, usually presents as a slowly growing, solitary mass that is detected at a later stage and often leads to tissue necrosis or dystrophic calcification due to inadequate blood supply caused by large tumors. Imaging findings of pancreatic FDCS can be similar to that of PNENs, GITS, solid pseudopapillary tumors (SPT), and pancreatic cancers, and cystic lesions can also occur, so FDCS should be considered when imaging shows a mass lesion of the pancreas with a complex internal structure. Due to the scarcity of systematic imaging studies of pancreatic FDCS, it becomes a more challenging task to diagnose this disease by imaging alone.

Histological findings and specific immunohistochemical staining are the gold standards for diagnosing FDCS. Tumor cells with spindle-shaped or oval morphology arranged in swirls and bundles, and binuclear or multinuclear morphology, are typical pathological features of FDCS [14]. In terms of immunophenotyping, CD21, CD23, and CD35 are the most diagnostic markers of FDCS [15]. CD21 and CD23 have been reported to show positive rates of 83% and 90%, respectively [7]. In 5 of the 7 previous cases of pancreatic FDCS, both CD21 and CD23 were expressed, which is useful for distinguishing it from other diseases such as cross-dendritic cell sarcoma (IDCS), Langerhans cell sarcoma (LCS), and histiosarcoma (HS) [16]. Wu et al. [17] analyzed 43 cases of mediastinal FDCS and reported an overall misdiagnosis rate of 83.3% in 18 patients who underwent preliminary needle biopsy. Our patient underwent two pancreatic punctures before surgery, but a clear diagnosis was not obtained. CT-guided puncture suggested myeloid sarcoma (MS) due to positive immunohistochemistry for MPO, CD45, and Ki-67 and negative CK, CD3, and CD20. However, in addition to positive MPO, no special MS markers including CD43, LCA, and lysozyme were detected in this sample, and given that isolated MS is extremely rare and bone marrow examination was normal, surgical pathology ultimately confirmed the diagnosis as FDCS. Clarifying the nature of the tumor in our patient was challenging, as both fine-needle aspirations failed to yield conclusive results. Combined with previous literature, it is speculated that the reasons are as follows. Firstly, pancreatic FDCS is a rare disease, which may not be considered by clinicians as a potential diagnosis. Secondly, imaging findings often overlap with those of more common pancreatic tumors, such as GITS and pNENs, leading to a lack of specificity. Thirdly, the tumor is often large with a complex internal composition, and the limited amount of specimen obtained via fine needle aspiration may not be sufficient to represent the entire tumor, particularly in cases where surrounding tissues have undergone unclear decomposition. Finally, the histological features of FDCS can be similar to those of other malignant tumors, making accurate diagnosis challenging. Given these factors, preoperative diagnosis of pancreatic FDCS is difficult, and external puncture may even increase the risk of misdiagnosis. Additionally, puncture of a blood-rich mass increases the risk of bleeding, which may be an important consideration in assessing the risks associated with this type of surgery [18]. Therefore, surgery is the preferred approach for obtaining valid specimens and confirming the diagnosis of FDCS.

The lack of prospective studies related to treatment has resulted in the absence of a definitive uniform standard for FDCS treatment. The CHOP regimen is currently the most widely used for patients who cannot undergo surgery or have widespread disease, but for most patients, radical surgery remains the preferred option. Surgical excision is both helpful in disease diagnosis and the most direct means of symptom relief. Fonseca et al. [19] conducted a study of 12 FDCS patients and found that the recurrence rates of surgery alone, chemoradiotherapy, and combination therapy were 40%, 56%, and 20%, respectively. This suggests that postoperative adjuvant chemoradiotherapy may be beneficial in improving patient prognosis, but the benefits of adjuvant therapy are still debated; some scholars proposed that adjuvant therapy does not provide obvious survival advantages for such patients [3]. It is now widely recognized that mesenchymal cells are the true precursor cells of FDCS origin [20], and this has been demonstrated in some cases of clinical remission after treatment with gemcitabine and taxane. Additionally, the overexpression of programmed death protein 1 (PD-1) and its associated ligands in FDCS presents a potential avenue for the treatment of FDCS [21]. Despite being classified as a low- to moderate-grade malignancy, FDCS is prone to recurrence and metastasis [22]. Studies have reported extranodal FDCS to have high rates of recurrence (42%), distant metastasis rates (21%), and local recurrence rates (21–23%). Long-term follow-up data for patients with FDCS are scarce due to the rarity of the disease. Of the seven cases reported, only one patient received adjuvant therapy, while the remaining six were treated with surgery alone, and outcomes varied. One patient died of postoperative lung infection, four had recurrent metastasis, and the remaining two did not report disease outcomes. Our patient underwent postoperative CHOP regimen chemotherapy and showed no recurrence after 11 months of follow-up.

This article comprehensively explores the imaging features, treatment, and prognosis of pancreatic FDCS, with a focus on analyzing the causes of misdiagnosis during puncture, providing a new understanding of the disease. However, there are also shortcomings, due to the rarity of this tumor; long-term follow-up and systematic observation have not been conducted, so further exploration is needed for standardized treatment of this disease.

## Conclusion

Diagnosing pancreatic FDCS can be challenging due to the lack of specificity in laboratory and imaging tests. The external puncture may lead to misdiagnosis; thus, surgery is the preferred method for both diagnosis and treatment, although the development of standardized treatment protocols requires further investigation. Moreover, predicting the behavior of FDCS tumors can be difficult, and the abdomen is a common location for FDCS, which is a poor prognostic factor. Especially when it occurs in rare retroperitoneal organs such as the pancreas, it is often misdiagnosed due to unrecognizability, so high vigilance should be exercised.

## Data Availability

All data generated or analyzed during this study are included in this published article.

## Abbreviations

FDCS: Follicular dendritic cell sarcoma
CT: Computed tomography
MRI: Magnetic resonance imaging
M: Male
F: Female
CD21: Cluster of differentiation 21
CD23: Cluster of differentiation 23
CD35: Cluster of differentiation 35
MS: Myeloid sarcoma
EUS: Endoscopic ultrasound
PET-CT: Positron emission computed tomography

## References

[1] Monda L, Warnke R, Rosai J (1986). A primary lymph node malignancy with features suggestive of dendritic reticulum cell differentiation. A report of 4 cases. Am J Pathol..

[2] Saygin C, Uzunaslan D, Ozguroglu M (2013). Dendritic cell sarcoma: a pooled analysis including 462 cases with presentation of our case series. Crit Rev Oncol Hematol..

[3] Gounder M, Desai V, Kuk D (2015). Impact of surgery, radiation and systemic therapy on the outcomes of patients with dendritic cell and histiocytic sarcomas. Eur J Cancer..

[4] Lu T, Song B, Pu H (2019). Paraneoplastic pemphigus and myasthenia gravis as the first manifestations of a rare case of pancreatic follicular dendritic cell sarcoma: CT findings and review of literature. BMC Gastroenterol..

[5] Hollowood K, Stamp G, Zouvani I (1995). Extranodal follicular dendritic cell sarcoma of the gastrointestinal tract. Morphologic, immunohistochemical and ultrastructural analysis of two cases. Am J Clin Pathol..

[6] Shen SC, Wu CC, Ng KF (2006). Follicular dendritic cell sarcoma mimicking giant cell carcinoma of the pancreas. Pathol Int..

[7] Soriano AO, Thompson MA, Admirand JH, et al. Follicular dendritic cell sarcoma: a report of 14 cases and a review of the literature. Am J Hematol. 2017;82:725–28. 10.1002/ajh.20852 17373675

[8] Liang W, He W, Li Z (2016). Extranodal follicular dendritic cell sarcoma originating in the pancreas: a case report. Medicine (Baltimore)..

[9] Mograbi M, Stump MS, Luyimbazi DT (2019). Pancreatic inflammatory pseudotumor-like follicular dendritic cell tumor. Case Rep Pathol..

[10] Lu X, Wu Y, Gong J (2022). Pancreatic follicular dendritic cell sarcoma: one case report and literature review. J Int Med Res..

[11] Mao S, Dong J, Wang Y (2021). Follicular dendritic cell sarcomas: CT and MRI findings in 20 patients. AJR Am J Roentgenol..

[12] Li J, Geng Z-J, Xie C-M, et al. Computer tomography imaging findings of abdominal follicular dendritic cell sarcoma. Medicine. 2016,95(1):e2404. 10.1097/MD.0000000000002404 PMC4706263 26735543

[13] Kang TW, Lee SJ, Song HJ (2010). Follicular dendritic cell sarcoma of the abdomen: the imaging findings. Korean J Radiol..

[14] Dalia S, Jaglal M, Chervenick P (2014). Clinicopathologic characteristics and outcomes of histiocytic and dendritic cell neoplasms: the moffitt cancer center experience over the last twenty five years. Cancers (Basel)..

[15] Shia J, Chen W, Tang LH (2006). Extranodal follicular dendritic cell sarcoma: clinical, pathologic, and histogenetic characteristics of an underrecognized disease entity. Virchows Arch..

[16] Yan WX, Yu YX, Zhang P (2019). Follicular dendritic cell sarcoma detected in hepatogastric ligament: a case report and review of the literature. World J Clin Cases..

[17] Wu YL, Wu F, Xu CP (2019). Mediastinal follicular dendritic cell sarcoma: a rare, potentially under-recognized, and often misdiagnosed disease. Diagn Pathol..

[18] Long-Hua Q, Qin X, Ya-Jia G (2011). Imaging findings of follicular dendritic cell sarcoma: report of four cases. Korean J Radiol..

[19] Fonseca R, Yamakawa M, Nakamura S (1998). Follicular dendritic cell sarcoma and interdigitating reticulum cell sarcoma: a review. Am J Hematol.

[20] Vermi W, Giurisato E, Lonardi S (2013). Ligand-dependent activation of EGFR in follicular dendritic cells sarcoma is sustained by local production of cognate ligands. Clin Cancer Res..

[21] Pardoll DM (2012). The blockade of immune checkpoints in cancer immunotherapy. Nat Rev Cancer..

[22] Li L, Shi YH, Guo ZJ (2010). Clinicopathological features and prognosis assessment of extranodal follicular dendritic cell sarcoma. World J Gastroenterol..

